# Dynamic DNA methylation landscape defines brown and white cell specificity during adipogenesis

**DOI:** 10.1016/j.molmet.2016.08.006

**Published:** 2016-08-17

**Authors:** Yen Ching Lim, Sook Yoong Chia, Shengnan Jin, Weiping Han, Chunming Ding, Lei Sun

**Affiliations:** 1 School of Laboratory Medicine and Life Science, Wenzhou Medical University, Wenzhou, Zhejiang 325035, China; 2 Cardiovascular and Metabolic Disorders Program, Duke-NUS Graduate Medical School, 8 College Road, Singapore 169857, Singapore; 3 Singapore Bioimaging Consortium, Agency for Science, Technology and Research (A*STAR), Singapore 138667, Singapore; 4 Institute of Molecular and Cell Biology, 61 Biopolis Drive, Proteos, Singapore 138673, Singapore

**Keywords:** Brown adipogenesis, White adipogenesis, DNA methylation, Hoxc10, Next generation sequencing

## Abstract

**Objective:**

DNA methylation may be a stable epigenetic contributor to defining fat cell lineage.

**Methods:**

We performed reduced representation bisulfite sequencing (RRBS) and RNA-seq to depict a genome-wide integrative view of the DNA methylome and transcriptome during brown and white adipogenesis.

**Results:**

Our analysis demonstrated that DNA methylation is a stable epigenetic signature for brown and white cell lineage before, during, and after differentiation. We identified 31 genes whose promoters were significantly differentially methylated between white and brown adipogenesis at all three time points of differentiation. Among them, five genes belong to the Hox family; their expression levels were anti-correlated with promoter methylation, suggesting a regulatory role of DNA methylation in transcription. Blocking DNA methylation with 5-Aza-cytidine increased the expression of these genes, with the most prominent effect on *Hoxc10*, a repressor of BAT marker expression.

**Conclusions:**

Our data suggest that DNA methylation may play an important role in lineage-specific development in adipocytes.

## Highlights

• DNA methylation is a stable epigenetic signature for brown and white adipocyte lineage.
• We identified 31 genes whose promoters were significantly differentially methylated between white and brown adipogenesis.
• Blocking DNA methylation with 5-Aza-cytidine increase the expression of a group of HoxC genes.

## 1 Introduction

Sedentary life style and frequent consumption of energy-dense food are primary causes for the escalating obesity rate. Obesity is a known risk factor for type 2 diabetes, cardiovascular diseases, hyperglycaemia, dyslipidaemia, hypertension, and cancers [1], [2], [3]. As such, understanding fat biology is a key area of interest. While white adipose tissues (WAT) function mainly as a primary organ for energy storage in the form of triglycerides to provide fuels during starvation, brown adipose tissues (BAT) serves as the major organ for non-shivering thermogenesis. Brown adipocytes are densely packed with uncoupled mitochondria expressing high levels of *Ucp1* that facilitates proton leak across the inner membrane to matrix, resulting in heat generation. Recent studies have also shown the existence of a third category of fat cells, known as beige or brite adipocytes. Dispersed among white adipose tissues, these beige adipocytes look indistinguishable from white adipocytes in the basal state but take morphological resemblance to BAT and express high levels of *Ucp1* when activated by β-adrenergic receptor agonist or proliferator-activated receptor-γ (*Ppar-γ*) [4], [5], [6]. This unique feature of brown and beige adipocytes make them attractive targets for obesity therapy [5], [6], [7], [8], which has been further bolstered by the recent re-discovery of BAT in adult human using positron emission tomography (PET) scans [9], [10], [11].

Elucidating molecular mechanisms underlying lineage-specific development in brown and white adipocytes is a topic of great interest. Recent years have seen significant progress in understanding the transcriptional regulatory circuits in these processes [12], but our knowledge about epigenetic regulation at DNA methylation level remains very limited. DNA methylation is one of the most extensively studied epigenetic mechanisms in a variety of biological processes including cell differentiation, cell type specificity, X chromosome inactivation, genomic imprinting, and development [13], [14]. Selective examination of methylation status of adipogenic markers (*Leptin*, *Pparg2*, *Fabp4* and *LPL*) and non-adipogenic makers (*MYOG*, *CD31* and *GAPDH*) in human mesenchymal stem cells, adipose tissue-derived stems cells, and embryonic stem cells showed that adipogenic promoters tend to be hypomethylated compared to non-adipogenic promoters, suggestive of an epigenetic control on the expression of adipogenic genes [15]. A previous study by Gentile et al. [16] reported that silencing *DNMT1* resulted in an acceleration of adipogenesis, which was accompanied by an apparent early induction of adipocyte-specific genes (*Glut4*, *Fabp4* and *PPARγ*). Adiponectin, a glucose metabolism regulator, was also found to be hypermethylated in promoter region of obese mice [17].

In this study, we seek to understand the epigenetic contribution of DNA methylation in controlling adipocyte differentiation and brown/white cell type definition. We comprehensively analyzed the DNA methylome of brown and white adipocytes during the course of differentiation. We found an overall hypermethylation in mature adipocytes compared with preadipocytes as well as in white adipocytes compared with brown adipocytes across the course of adipogenesis. Comparative analysis of brown vs. white methylome reveals differential methylation states of a set of *Hox* genes, whose mRNA levels are depot specific and likely regulated by DNA methylation.

## 2 Results

### 2.1 An overview of DNA methylation profiles for three adipogenesis models

**Figure 1:**
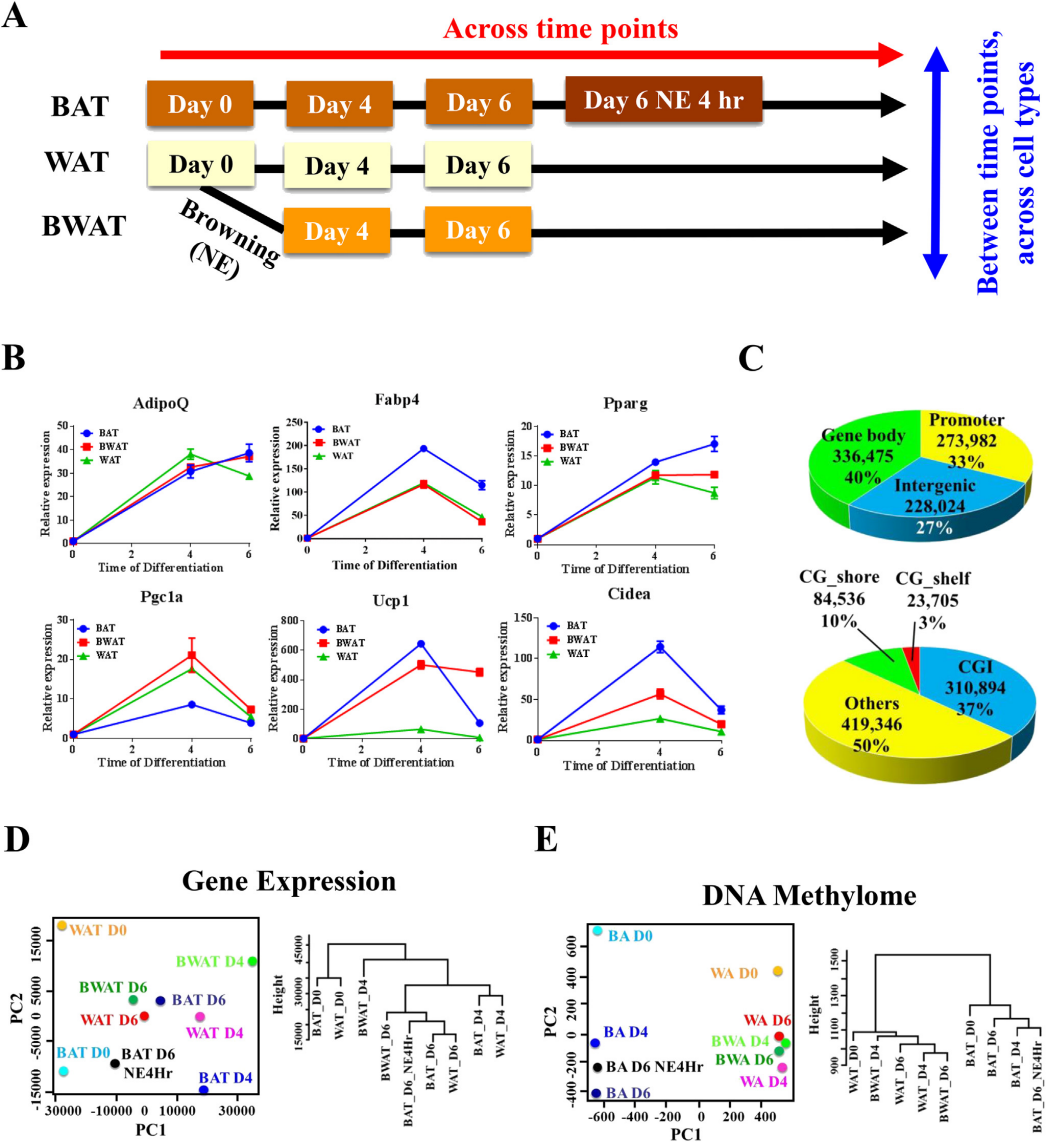
**Data overview**. (A) Study Design. BA: Brown adipocytes, WA: white adipocytes, BWA: brown induced white adipocytes. (B) Real-time quantitative PCR analyses of gene expression profiles for general adipogenic markers (top panel: AdipoQ, Fabp4 and Pparγ) and brown fat specific markers (bottom panel: Pgc1α, Ucp1 and Cidea) during brown, white, and brown-induced adipogenesis. Error bars represent mean ± SEM, n = 3. BAT: brown adipocytes, WAT: white adipocytes, BWAT: brown induced white adipocytes. (C) A total of 838,481 autosomal CpGs with sequencing depth of ≥10 for all nine samples were included in all downstream analyses. Covered CpGs were approximately uniformly distributed in promoters, gene bodies, and intergenic regions. 50% of the CpGs were located within 6 kb from annotated CGIs (CGI, CG shelf and CG shore). (D) PCA and hierarchical clustering analyses of all nine analyzed samples using gene expression from RNA-seq separated samples by states of differentiation. (E) PCA and hierarchical clustering analyses of all nine analyzed samples using DNA methylation separated samples by cell types. Average DNA methylation percentage generated by merging nearby CpGs of less than 500 bp was used.

To profile the DNA methylome for lineage specific adipogenesis, we isolated primary murine brown and white pre-adipocytes from interscapular and inguinal depots, respectively, for *in vitro* differentiation to mature adipocytes as described in the Methods. To examine methylome changes during browning and BAT activation, we also treated white adipocytes with norepinerephine (NE) for five days and acutely treated brown adipocytes with NE for 4 h to induce the expression of key BAT markers (Figure 1A). The biological validity of each model was confirmed by real-time quantitative PCR to examine the expression of the pan-adipogenic markers (*AdipoQ, Fabp4, Pparγ*) and BAT-specific markers (*Pgc1*α*, Ucp1, Cidea*) across the differentiation stages (Figure 1B).

Each of these nine samples listed in Figure 1A was subjected to a modified reduced representation bisulfite sequencing (RRBS) pipeline developed in-house [18] to quantify DNA methylation. Using a minimum sequencing depth of 10 as a cut off, we obtained an average of 1.3 million autosomal CpGs per sample with a minimal conversion rate at 99.2% (Figure S1A–S1B). To facilitate comparison, only CpGs with ≥10 coverage in all nine samples were retained for downstream analysis. This reduced the pool of analyzable autosomal CpGs to 838,481. Similar to previous study in human placenta [19], 33%, 40% and 33% of these sites were distributed in promoters (defined as 1 kb upstream and 500 bp from TSS, 287,243 CpGs, 33%), gene bodies (defined as from end of promoter to TTS, 315,733 CpGs, 40%) and intergenic regions (27%) (Figure 1C top panel). About half of the CpGs were located within 6 kb of annotated CpG island (CGIs), comprising of CGIs (37%), CG shores (2 kb up/downstream from CGI, 10%), and CG shelves (2 kb up/downstream from CG shores, 3%) (Figure 1C bottom panel). On a regional level, 47.5% of promoters, 56.1% of gene bodies and 63.8% of CGIs are covered by at least two CpGs (Figure 1C). Out of the 838,481 CpGs, 122,818 CpGs (14.7%) were mapped to repetitive elements (Figure S1C).

To ask what biological information the DNA methylome and transcriptome may encode, we performed principle component analysis and hierarchical clustering to both datasets. Clustering with transcriptome data revealed separation of samples by their states of differentiation (Figure 1D) associated with a functional specialization towards lipid metabolism (Figure S3A). However, DNA methylomes grouped samples by cell types regardless their differentiation status (Figure 1E). Noteworthy, NE treatment in WAT and BAT did not change their clustering pattern based on DNA methylation. Therefore, our results demonstrated that DNA methylation signature represents a stable epigenetic mark for different cell lineages.

### 2.2 Overall hypermethylation during adipogenesis

**Figure 2:**
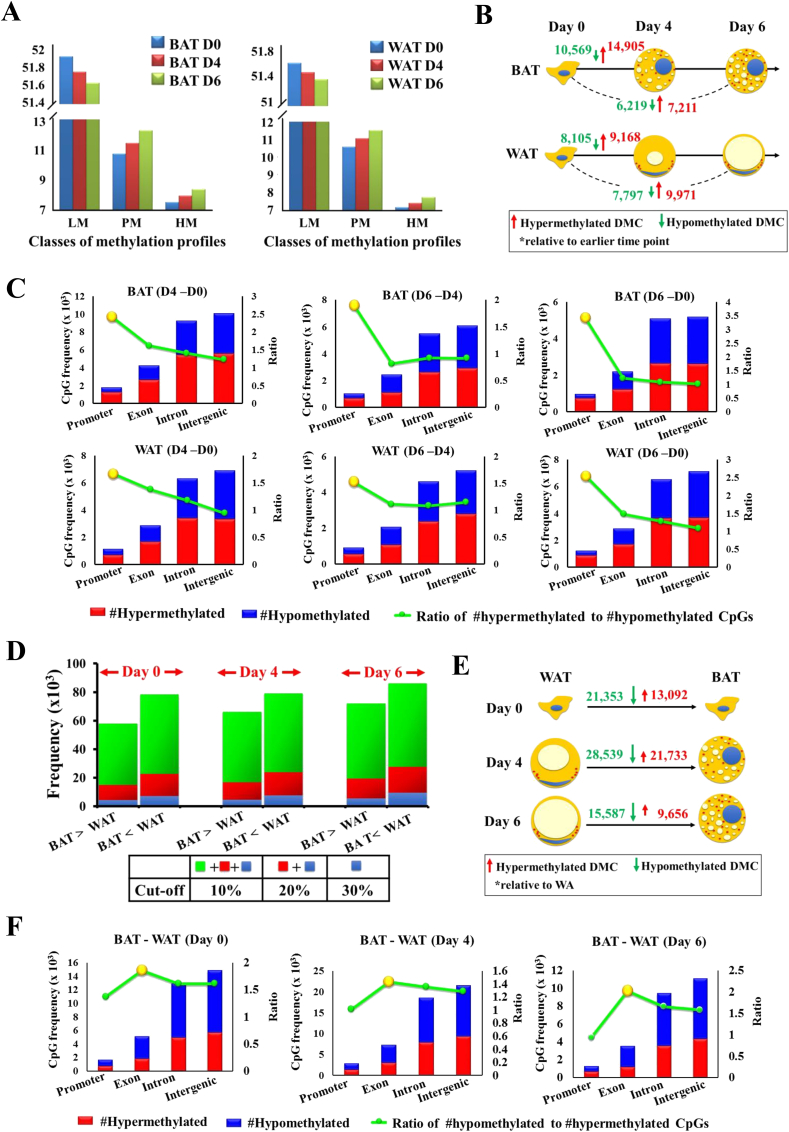
**Overall hypermethylation during adipogenesis and hypermethylation in white adipocytes than brown adipocytes**. (A) Individual CpGs grouped by methylation levels into LM (<30%), PM (30–70%) and HM (>70%) during brown (left panel) and white (right panel) adipogenesis. (B) Frequency of DMCs during brown and white adipogenesis. Comparisons were made relative to the earlier time point (i.e. day 4 – day 0, day 6 – day 0). Green arrow: hypomethylation. Red arrow: hypermethylation. (C) Proportions of significantly differentially methylated CpGs in each genomic category. DMCs were mostly located in intron and intergenic regions. Ratio between hyper- and hypomethylated DMCs given by line plot. (D) Frequencies of hyper- and hypomethylated CpGs between BAT and WAT at various days of differentiation (day 0, 4 and 6), using varying cutoffs of 10%, 20%, and 30%. (E) Frequency of DMCs during with respect to WAT at days 0, 4, and 6. Green arrow: hypomethylation. Red arrow: hypermethylation. (F) Proportions of significant differentially methylated CpGs in each genomic category. DMCs were mostly located in intron and intergenic regions. Ratio between hyper- and hypomethylated DMCs given by line plot.

To determine how the DNA methylome changes during adipogenesis, we examined DNA methylation profile changes during the time course of differentiation at single CpG and genomic region levels. First, all analyzed CpGs were categorized into lowly methylated (LM, <30%), partially methylated (PM, 30–70%) and highly methylated (HM, >70%) classes. During both white and brown adipogenesis, there was a decrease in LM CpGs and a corresponding increase of PM and HM CpGs (Figure 2A). Further assessment of methylation difference for each CpG revealed a consistent higher abundance of hyper- than hypomethylated CpGs relative to an earlier time point (Figure 2A, Figure S2A). These data suggested an overall increase in DNA methylation during both white and brown adipogenesis. Interestingly, although most of the significantly differentially methylated CpGs (DMC) (Figure 2B) were located in intronic and intergenic regions, dominance of hypermethylation was most pronounced in promoter regions (Figure 2C). Independent functional enrichment analyses on significantly differentially methylated promoters (DMP) of brown and white adipogenesis (day 0 vs day 4) showed significant enrichments in cell proliferation and differentiation (Figure S2B).

### 2.3 White adipocytes exhibit consistent overall hypermethylation compared to brown adipocytes during all differentiation stages

Intrigued by cell lineage specificity of the DNA methylome from Figure 1F, we proceeded to investigate the systematic differences between BAT and WAT throughout adipogenesis. Time-matched comparisons between BAT and WAT revealed hypermethylated CpGs significantly outnumbered hypomethylated CpGs (p < 0.05, binomial test, Figure 2D,E). Similar to findings from adipogenesis time course analysis, significantly differentially methylated CpGs (DMCs) (Figure 2F) were mostly located in intronic or intergenic regions. However, unlike in adipogenesis time course analysis, dominance of hypomethylated DMCs (with respect to WAT) was found in exonic regions instead of promoters (Figure 2F). In addition, significantly differentially methylated promoters (DMPs) between mature BAT and WAT showed enrichment for networks related to brown fat functions (Figure S2C).

### 2.4 General anti-correlation between promoter methylation and gene expression

To investigate if changes in promoter DNA methylation were associated with gene expression changes, we used transcriptome data obtained from sample-matched RNA-seq data. Global gene ontology analyses on the regulated genes during white and brown adipogenesis showed significant enrichments in mitochondrial respiratory chain and lipid metabolic process terms respectively (Figure S3A), whereas top up-regulated GO in mature BAT relative to mature WAT includes mitochondrial respiratory chain and fatty acid oxidation (Figure S3B). This clearly demonstrated that our RNA-seq data accurately reflects biological changes during differentiation and between different cell types. An overall negative correlation between promoter methylation and gene expression was observed when comparing WAT and BAT types (Figure S4A) and cells at different time course of adipogenesis (Figure S4B).

### 2.5 Hox family genes were consistently differentially methylated between brown and white adipogenesis at all time points

**Figure 3:**
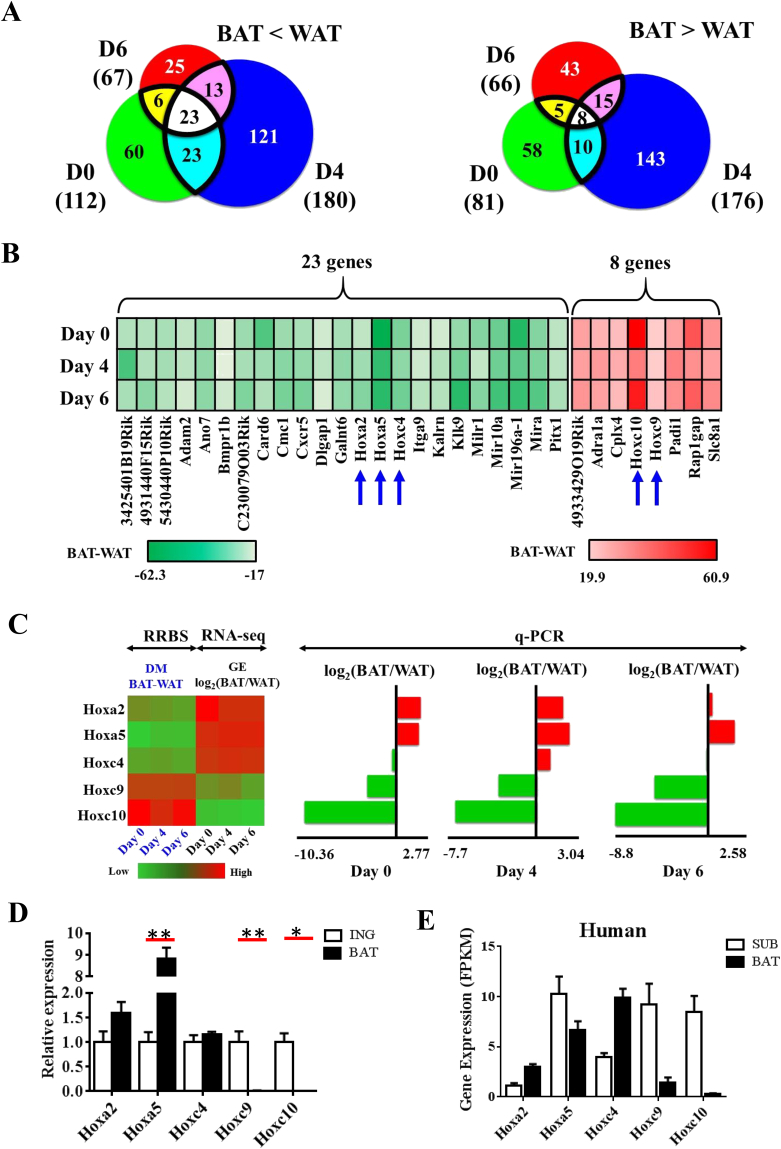
**Promoter methylation differences for five Hox genes were associated with gene expression differences between white and brown adipocytes during adipogenesis**. (A) Frequencies of overlapping DMPs between BAT and WAT at days 0, 4, and 6. DMPs were split into BAT > WAT and BAT < WAT. (B) Heatmap representation of methylation differences for gene promoters (between BAT and WAT) which were either consistently hypo or hypermethylated from day 0–6 of cell differentiation. Five *Hox* genes have been indicated by blue arrows. (C) Promoter DNA methylation difference (RRBS) with corresponding gene expression (RNA-seq) difference between WAT and BAT at days 0, 4, and 6 for five selected *Hox* genes (*Hoxa2, Hoxa5, Hoxc4, Hoxc9*, *and Hoxc10*). Gene expression difference for five selected *Hox* genes were validated with q-PCR results. (D) Expression of the five *Hox* genes in mouse inguinal and brown tissues. Error bars represent mean ± SEM, n = 4. *p ≤ 0.05, **p ≤ 0.01. (E) RNA-seq expression of the five *Hox* genes in human subcutaneous and brown tissues. Error bars represent mean ± SEM, n = 3/4.

We identified 31 genes with significant changes in DNA methylation when comparing BAT vs. WAT at all three time points during adipogenesis (Figure 3A). Out of these 31 genes (Figure 3B), 23 and 8 were hyper- and hypomethylated, respectively, in WAT compared to BAT. There was a significant enrichment of an important class of transcription factors, Hox family genes, including *Hoxa2*, *Hoxa5*, *Hoxc4*, *Hoxc9* and *Hoxc10* (p < 0.01, hypergeometric test). Hox gene members are a super family of transcription factors [20], which are highly conservative in sequence and functions. Recent work has demonstrated the involvement of Hox genes in the adipogenic field such as cell specific expression and involvement in metabolic diseases such as diabetes [20], [21]. For instance, *Hoxc9*, hypermethylated in BAT, is a well-established white fat marker [22] (Figure 3B). *Hoxc8* was highly expressed in human WAT progenitor cells to repress brown fat markers [23].

Interestingly, for all these five *Hox* genes, gene expression by RNA-seq inversely correlates with the methylation pattern. *Hoxa2*, *Hoxa5* and *Hoxc4* were enriched in BAT, consistent with their hyper-methylation, while the expression of *Hoxc9* and *Hoxc10* was lower in BAT, consistent with their hypo-methylation. Furthermore, the relative expressions of these genes were confirmed by q-PCR. *Hoxa2*, *Hoxa5*, *Hoxc9* and *Hoxc10* showed consistent anti-correlation between methylation and gene expression changes (Figure 3C, right panel). Additionally, we analyzed the relative expressions of these *Hox* genes in mice tissue samples and observed a similar pattern as the *in vitro* model (Figure 3D).

To test whether the expression pattern of these genes is conserved between mouse and human, we examined RNA-seq data obtained from human adult subcutaneous and fetal brown adipose fats (Figure 3E). Consistent with both mouse *in vitro* and *in vivo* models, *Hoxc9* and *Hoxc10* exhibited tissue specific expression in human.

### 2.6 Inhibition of DNA methylation in Hox gene promoters by 5-Aza-cytidine treatment up regulates gene expression

**Figure 4:**
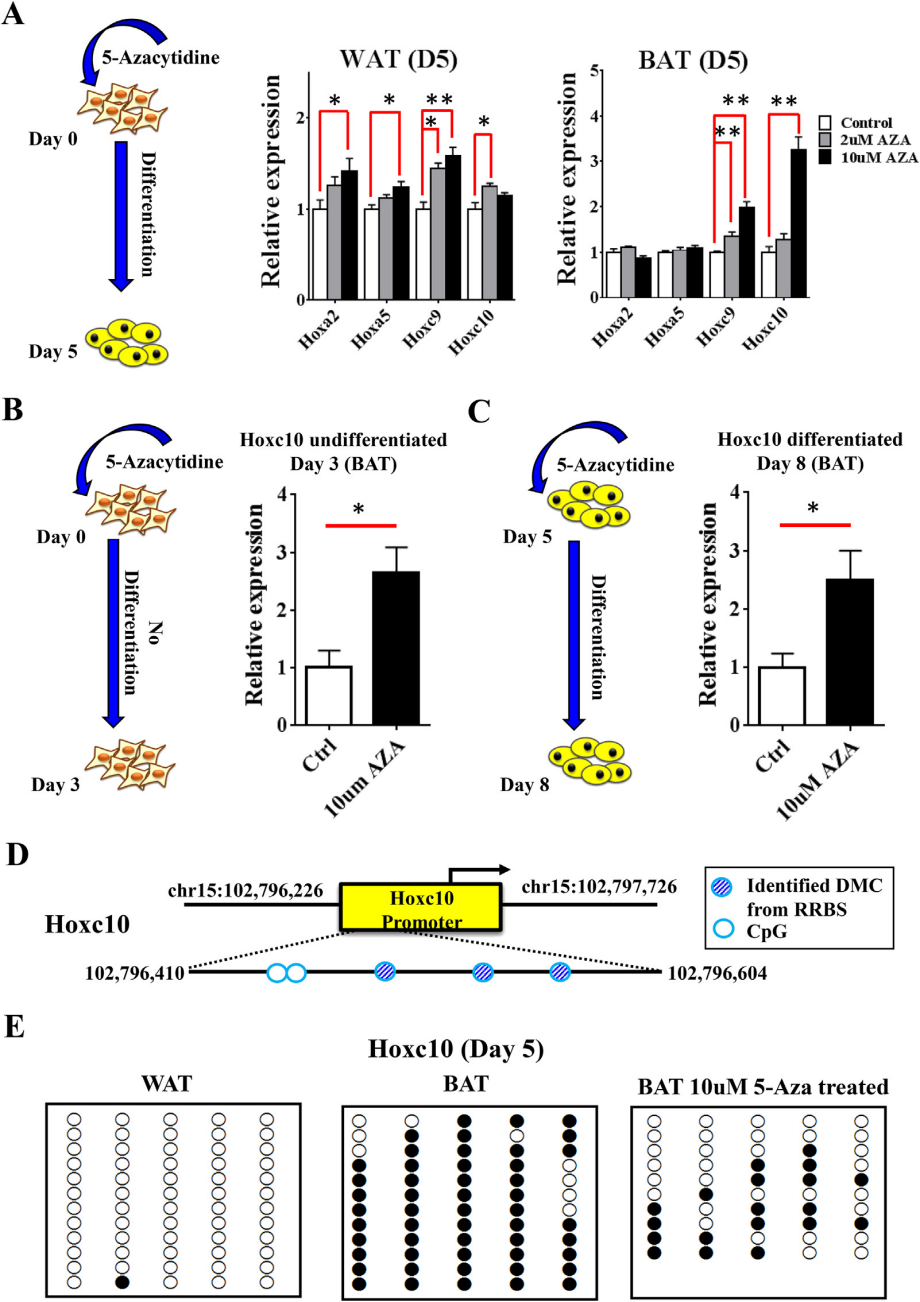
**Inhibition of DNA methylation in Hox gene promoters by 5 Aza-cytidine treatment alters gene expression**. (A) Schematic diagram of 5-Aza-cytidine treatment to BAT and WAT pre-adipocytes (left panel). Expression of Hox genes subjected to 2 μm and 10 μm of 5-Aza-cytidine treatment in BAT middle panel) and WAT (right panel) at day 5. There was a prominent elevation of *Hoxc10* gene expression in BAT brought by 10 μm 5-Aza. n = 4. (B–C) Brown pre-adipocytes were treated with 10 μm 5-Aza-cytidine and expression of Hoxc10 investigated under different differentiation conditions. (B) Undifferentiated BAT at day 3. (C) Cells treated at day 5 and harvested at day 8. Cells induced to differentiate and harvested at day 8. n = 3/4. (D) Selected *Hoxc10* promoter region for further analysis. This region spans 195bp, covering five CpG sites. Striped blue circles: CpG with significant methylation difference identified from RRBS. Open blue circles: CpG not covered in RRBS study. (E) Bisulfite treatment, cloning, and sequencing performed on white (WAT) and brown (BAT) adipocytes at day 5 in *Hoxc10* promoter confirmed hypermethylation status in BAT (relative to WAT). Bisulfite sequencing of the CpG sites before (BAT) and after 5-Aza-cytidine treatment (BAT 10 μm 5-Aza treated). Each open circle represents unmethylated cytosine and closed circle represents methylated cytosine. *p ≤ 0.05, **p ≤ 0.01.

Next, we asked if there is a potential causative relationship between promoter DNA methylation and gene expression for the *Hox* genes. We induced both white and brown pre-adipocytes to differentiate for five days in the presence of 5-Aza-cytidine, a blocker of methylation. Upon 5-Aza-induced demethylation, *Hoxa2* and *Hoxa5* were significantly upregulated in WAT, where their promoters were hypermethylated, but not in BAT, where their promoters were lowly methylated. *Hoxc9* and *Hoxc10* were up regulated more in BAT than in WAT, probably due to their hyper-methylation in BAT (Figure 4A). Hoxc10 was of particular interest and chosen for further analysis, because it manifested the most significant change in expression upon de-methylation, and our recent data have demonstrated that it is a repressor for BAT marker expression in WAT, which will be reported in a separate study.

Because 5-Aza treatment causes global demethylation, it is possible the observed changes in gene expression are due to an indirect effect. To exclude this possibility, we treated preadipocytes with 5-Aza for 3 days without adding the differentiation cocktail (Figure 4B), and, in a separate experiment, treated the differentiated mature brown adipocytes (D5) (Figure 4C) with 5-Aza for three days. Up-regulation of *Hoxc10* was detected in both cases, which argues that the up regulation of *Hoxc10* by demethylation is likely to be a causative effect and independent of the differentiation process.

To provide a precise picture of the methylation status in *Hoxc10* promoter at individual CpG level, we performed bisulfite conversion and cloned *Hoxc10* promoter region for sequencing (Figure 4D). Consistently with our high throughout sequencing data, methylation was only found in BAT but not WAT. Upon 5-Aza treatment, more than 60% of the CpG sites were unmethylated (Figure 4E), which was accompanied by an up-regulation of *Hoxc10* expression (Figure 4A–C). Taken together, our study demonstrated that BAT-specific methylation of *Hoxc10* promoter contributes to its tissue-specific gene expression repression.

## 3 Discussion

As one of the most well-studied epigenetic mechanisms, DNA methylation has been shown to be important in regulating cell differentiation [15], [24] and for defining cell lineages. Here, we present the first comprehensive and dynamic genome-wide DNA methylation landscape for both white and brown adipogenesis processes, to understand how fat cells differentiation and cell fate commitment are regulated by DNA methylation.

While gene expression profiles represent the dynamic status during cell differentiation in both BAT and WAT adipogenic models, DNA methylation profiles are consistently reflective of cell lineages. This suggests that while DNA methylation is known to regulate gene expression, its function may be better appreciated in development. Surprisingly, only 31 genes were consistently differentially methylated between BAT and WAT in the entire differentiation process. Five out of these 31 genes are *Hox* family genes that encode transcription factors that bind to DNA enhancers via homeodomain to either activate or suppress gene expression. We observed a similar differential expression pattern for *Hoxc10* in mouse primary cell lines and tissues as well as in human tissues. We also demonstrated that promoter methylation may have a direct effect in regulating *Hoxc10* expression.

Among many essential roles of the Hox genes include proper embryo development and control of cell death and cell proliferation [20], [25]. A recent study by Benton et al. [26] compared methylation changes before and after gastric bypass and weight loss human subcutaneous adipose and found significant methylation changes in *Hox* genes from multiple *Hox* clusters, suggesting the change in methylation of *Hoxc10* is clinically relevant. Collectively, our work suggests a potential pathway for how DNA methylation regulates adipogenesis via cell lineage commitment. This serves as an important and solid basis for supporting the role of DNA methylation being a stable epigenetic signature in defining brown and brown adipocyte lineage.

One limitation of our study is that brown and white preadipocytes were isolated from different fat depots. We can't preclude the possibility that the observed methylation difference between BAT and WAT might be related to their distinct localization. Studies to compare interscapular WAT covering BAT with BAT should be explored in the future.

## 4 Experimental procedures

### 4.1 Cell isolation and cell culture

Brown and white preadipocytes were isolated from interscapular brown adipose tissues and inguinal white fat depots respectively. Eight BL6 mice were sacrificed at three weeks and the harvested fat tissues were minced, collagenase digested, and fractionated. Pre-adipocytes, which were enriched at the bottom stromal vascular fractions, were collected, resuspended, and cultured to confluence. Subsequently, white and brown pre-adipocytes were induced to differentiate into mature brown and white adipocytes by exposure to differentiation medium: 10% (v/v) FBS DMEM, 850 nM insulin (Sigma), 0.5 μM dexamethasone (Sigma), 250 μM 3-isobutyl-1-methylxanthine (Sigma), and 1 μm Rosiglitazone (Sigma). 48 h later, we replaced the induction medium with maintenance medium (10% FBS, DMEM). In addition, an independent set of white pre-adipocytes was induced to differentiation in the presence of norepinephrine (1uM). Cells were harvested at days 0, 4 and 6 for both RRBS and RNA-seq library preparation.

### 4.2 Reduced representation bisulfite sequencing

A total of nine samples were included in the study. Experimental and computational procedures followed previously described methods [18], [19] with some modifications. Briefly, 1 μg of genomic DNA from each sample was digested using MspI (New England Biolabs, USA) and Taq^α^I (New England Biolabs). The digested product was subsequently purified with the QIAquick PCR Purification Kit (QIAGEN GmbH), end-repaired, 3′-end-adenylated, and adapter-ligated using TruSeq ChIP-Seq Sample Preparation Kit (Illumina, USA). Ten microliters of the RNA adapter index adapter oligonucleotides and 3 μL of ligation mix were used in a 45 μL reaction system and the ligation was performed for 10 min at 30 °C in the adapter-ligation step. Fragments with 210–290 bp size were selected by gel electrophoresis, purified, and bisulfite treated using EZ DNA Methylation-Gold Kit (Zymo Research, USA). The bisulfite converted DNA was then PCR amplified, purified by by Agencourt AMPure XP Beads (Beckman Coulter Inc.) and validated using Agilent 2100 Bioanalyzer (Agilent Technologies, Santa Clara, CA, USA). Libraries were pooled together and analyzed by paired-end sequencing (2 × 75 bp) read on Illumina Hiseq (High Output Mode). The mouse July 2007 (NCBI37/mm9) genome assembly was used throughout the study. Sequencing data were deposited into the GEO database with accession number GSE80961.

### 4.3 Differential DNA methylation analysis

Differential methylation analysis was performed at both the single CpG and regional levels. Only autosome CpGs (sequencing depth ≥10) common to all nine samples were included in all subsequent analyses. Between a pair of samples, only CpGs having a methylation difference of at least 10% were selected for 2-sided fisher exact test. P values were then adjusted by the Benjamini Hochberg method. A CpG was considered significant if (i) the difference between the sample pair was at least 10%, and (ii) the FDR corrected p value < 0.05. A promoter was considered significantly differentially methylated if it contained at least two significant CpGs, both of which must be regulated in the same direction (either all hypermethylated or hypomethylated). All statistical analyses were performed using R package.

### 4.4 RNA-seq

Total RNAs from adipocytes were extracted according to Qiagen miRNeasy kit. RNA-seq libraries were prepared according to NEBNext Ultra Directional RNA Library Prep Kit for Illumina and ran on Hiseq2000 sequencer platform. The 100 bp paired-end reads were first quality checked with FastQC (http://www.bioinformatics.babraham.ac.uk/projects/fastqc/), subsequently aligned to mm9, using Tophat (version tophat-2.0.11). The aligned reads were then put into Cufflinks (Version 2.1.1) to quantify gene expression into units known as fragments per kilobase of exon per million fragments mapped (FPKM). Genes with low expression (FPKM < 1 in all samples) were removed, leaving 14,491 autosomal genes. Sequencing data were deposited into the GEO database with accession number GSE80961.

### 4.5 q-PCR

RNA was reverse transcribed into cDNA with random primers (SuperScript II Reverse Transcriptase, Invitrogen), followed by PCR amplification using gene specific primers. Sybr Green based qPCR was performed in an Applied Biosystems 7900HT Fast Real-time PCR System, using RPL23 as an internal control for normalization. Data were analyzed by the relative quantification (ΔΔ*C*_t_) method.

### 4.6 5-Aza-cytidine drug treatment

5-Aza-cytidine treatment was administered to brown and white adipocytes under three different conditions. (i) Confluent preadipocytes were induced to differentiate, and differentiating cells were incubated with 0, 2 μM and 10 μM 5-Aza-cytidine for five days. (ii) Confluent brown preadipocytes were treated with 0 μM and 10 μM 5-Aza-cytidine for three days without differentiation induction. (iii) Differentiated brown adipocytes (day 5) were treated with 0 μM and 10 μM 5-Aza-cytidine for three days.

### 4.7 Cloning and bisulfite sequencing

Total genomic DNA was isolated using QIAamp DNA Micro Kit (Qiagen). Bisulfite conversion was performed with 1 μg of the genomic DNA using EZ DNA Methylation-Gold Kit (Zymo Research), according to manufacturer's instructions. Bisulfite converted DNA was then amplified in the Hoxc10 promoter region using the following set of primers: forward, TTAAAAGTAAGTTGAAGTTTTTGTTTTTTTGTTTTT; reverse CCTCCTTTACCCAAAATCCCTTAAATA (Table S1). The PCR products were then TA-cloned into pGEM-T Easy vector (Promega, Madison, WI, USA), and positive clones with inserts were sequenced.

### 4.8 Availability of data and material

Sequencing data for DNA methylation and RNA-seq are submitted into the GEO database with accession number GSE80961.

## Funding

This work was supported by Singapore NRF fellowship (NRF-2011NRF-NRFF 001-025) to L.S. This research is also supported by the Singapore National Research Foundation under its CBRG grant (NMRC/CBRG/0070/2014 and NMRC/CBRG/0101/2016) and administrated by the Singapore Ministry of Health's National Medical Research Council.

## Author contributions

C.D. and L.S. and W.H. conceived and designed the experiment. S.Y.C. and S.J. performed the experiments. Y.C.L. performed the bioinformatics analysis. C.D., L.S., and Y.C.L. wrote the manuscript.

## Appendix A Supplementary data

Table S1 Sequences of quantitative RT-PCR primers for candidate Hox genes and primer for bisulfite sequencing assay for Hoxc10. Table S1

Figure S1. **Descriptive statistics for covered CpGs in RRBS**. (A) For each sample, red bars represent the number of uniquely aligned reads, while blue bars are the non-uniquely or unaligned reads. The blue line gives the bisulfite rate for each sample. (B) The histogram gives the average number of CpGs sites for nine samples, with varying minimum sequencing depths. Error bars represents standard deviation for nine samples. (C) Combined percentage of LINE, SINE and LTR made up 78% of all 122,818 CpGs mapped to repetitive elements.

Figure S2. (A) Frequencies of hyper- and hypomethylated CpGs between BA and WA at various days of differentiation (day 0, 4, and 6), using varying cutoffs of 10%, 20%, and 30%. (B) Gene ontology analyses on the hypermethylated DMPs from day 0–4 for WA (left panel) and BA (right panel). DMPs were enriched for cell proliferation and cell differentiation in both white and brown adipogenesis. (C) Two out of top five regulatory network using DMPs between BA and WA at day 6 relative to day 0 showed functional relevance to brown adipocytes functions, (left panel) Cancer, skeletal and Muscular disorders, Tissue morphology and (right panel) Energy Production, Lipid Metabolism, Small molecule biochemistry. Genes identified in DMP list have been boxed in red.

Figure S3. (A) Gene set enrichment analysis on up-regulated genes during brown adipogenesis and white adipogenesis using RNA-seq data (Day 0 vs Day 6). (B) Gene set enrichment analysis showed that genes up-regulated in BAT compared to WAT at day 6 were enriched in mitochondrial respiratory chain and fatty acid oxidation.

Figure S4. (A) Correlation of promoter methylation changes with gene expression changes between BAT and WAT. (B) Correlation of promoter methylation changes with gene expression changes between during adipogenesis.
