# Supplementary figures and images for: Dynamic DNA methylation landscape defines brown and white cell specificity during adipogenesis
Source: Mol Metab. 2016 Aug 17;5(10):1033–41. doi: 10.1016/j.molmet.2016.08.006 (PMC5034609; doi:10.1016/j.molmet.2016.08.006)

A

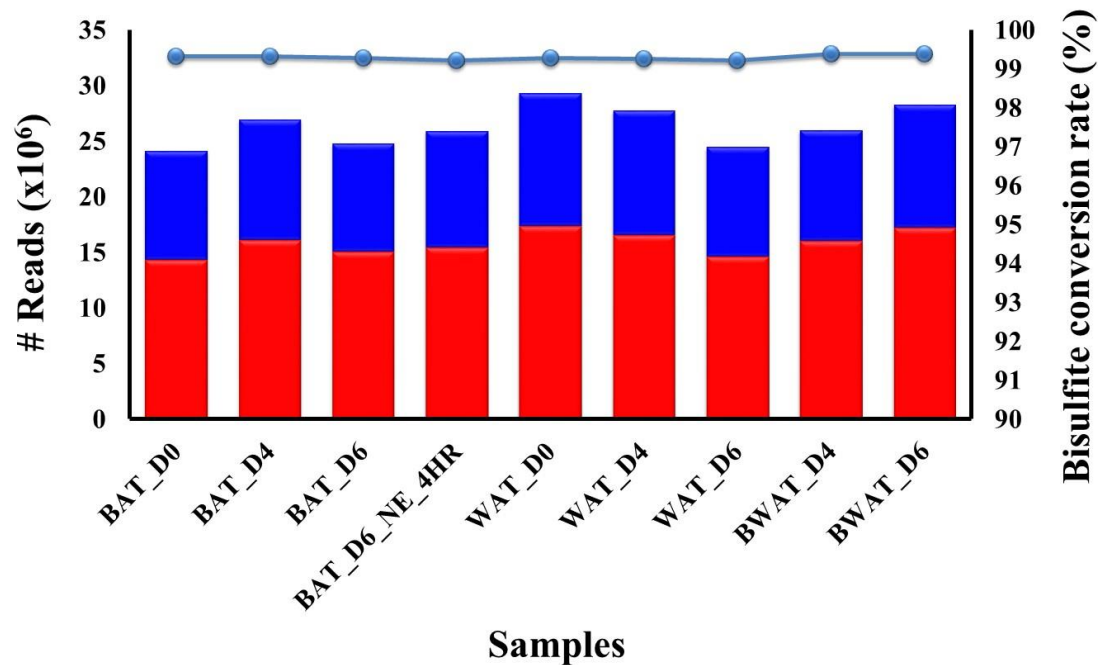

B

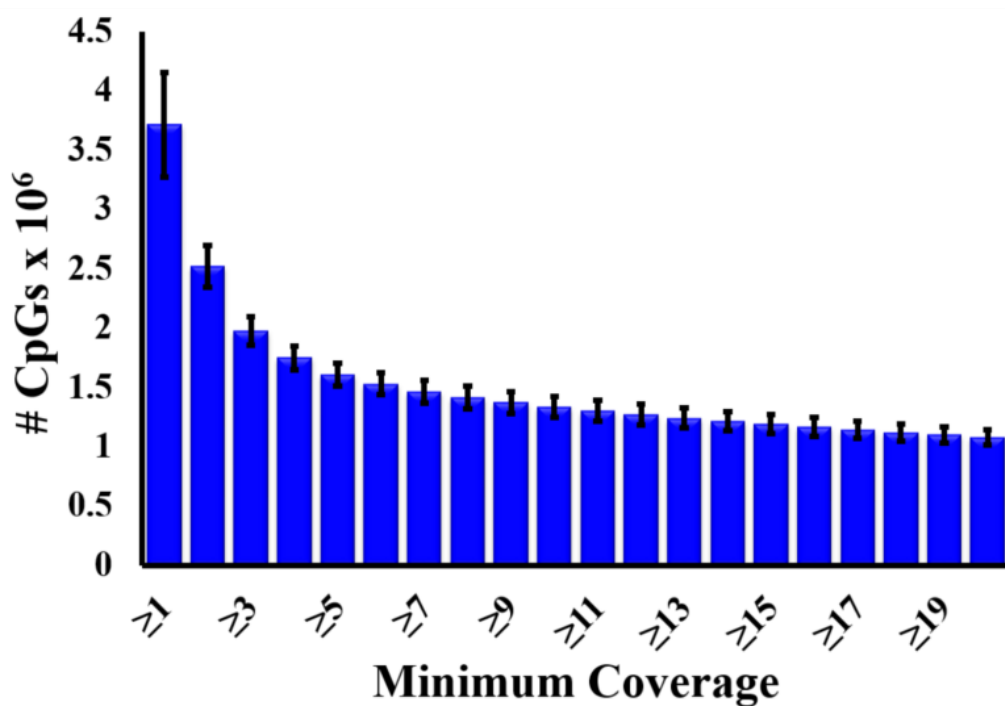

C

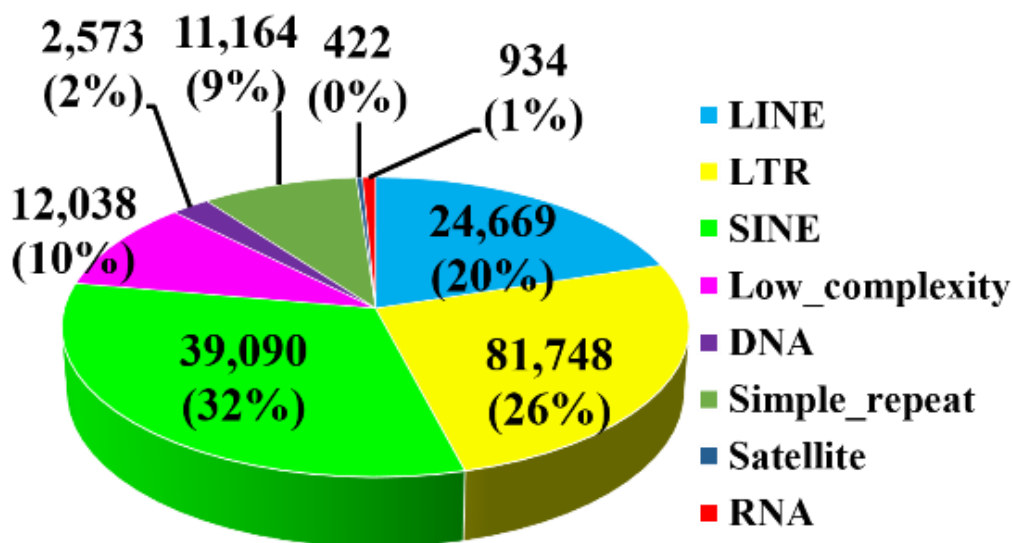

Figure S1

**A**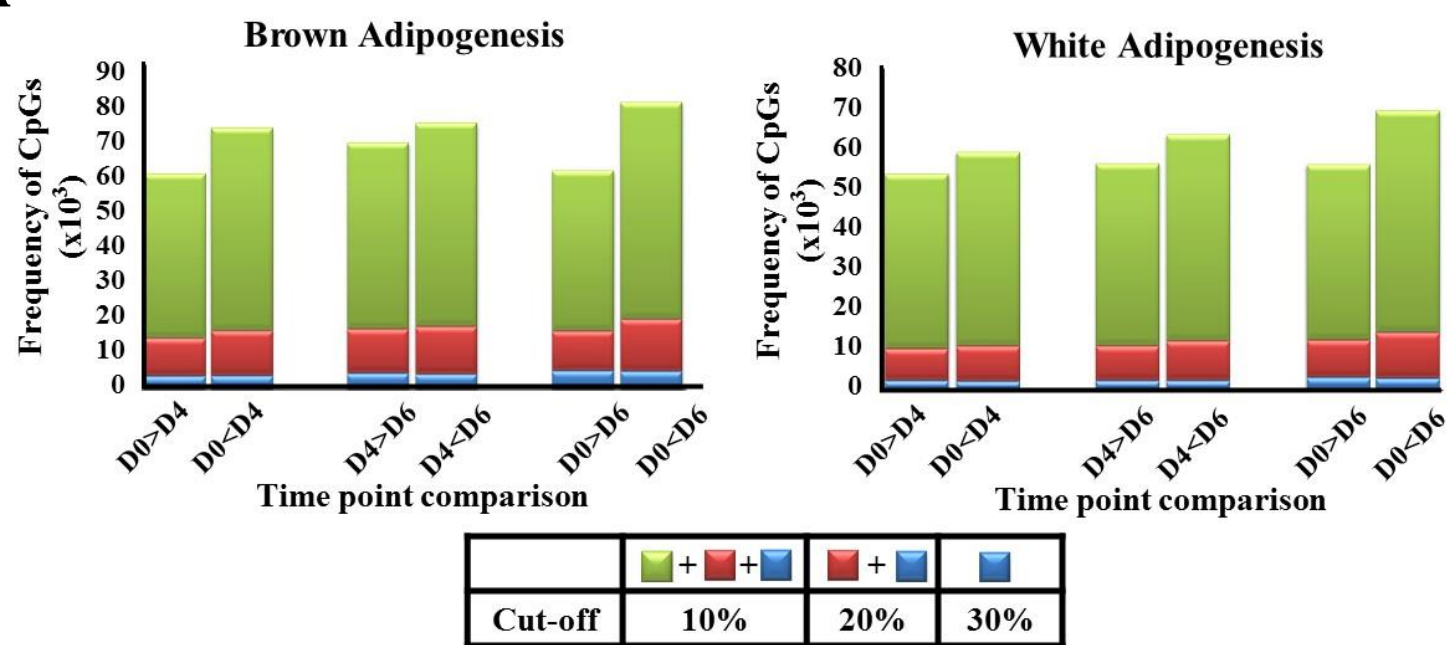**B**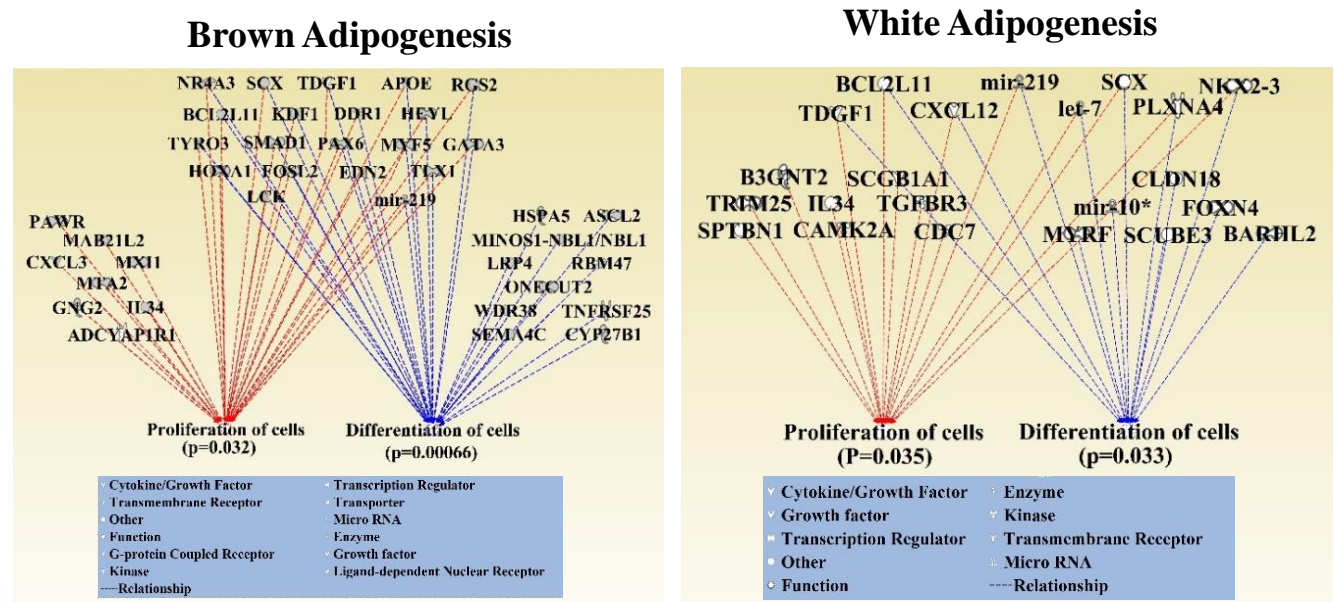**C**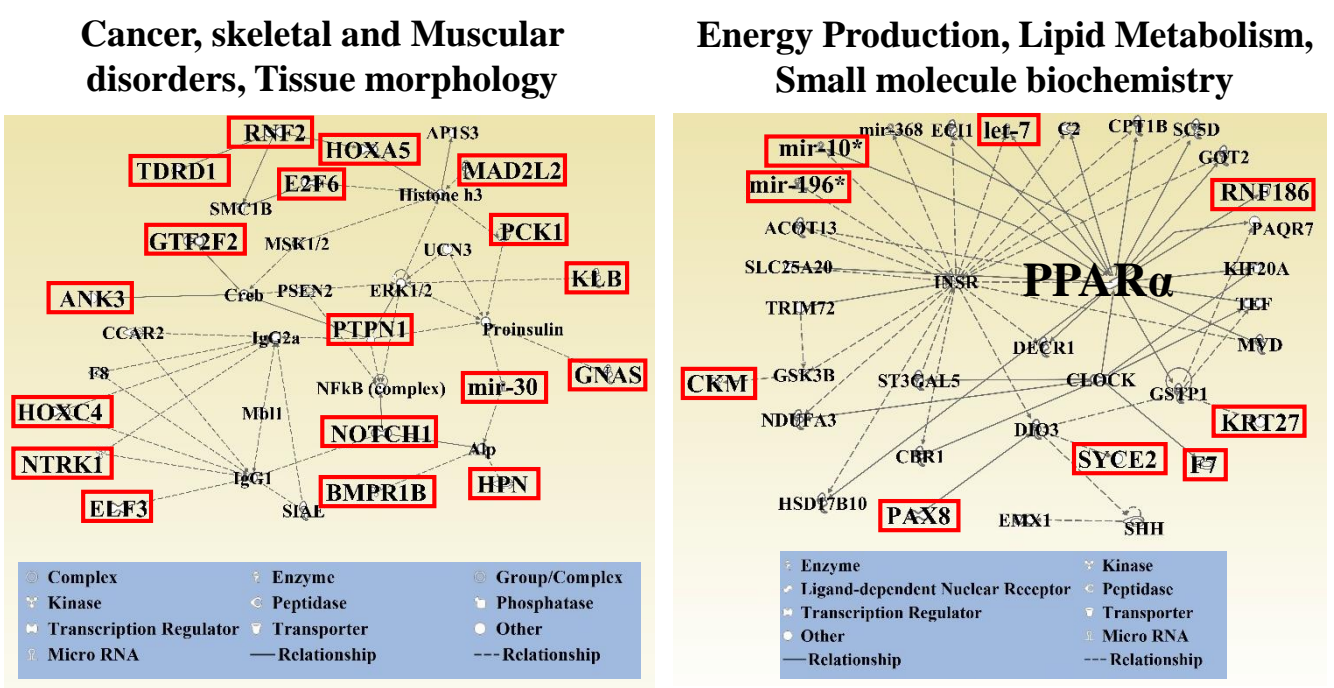**Figure S2**

**A**

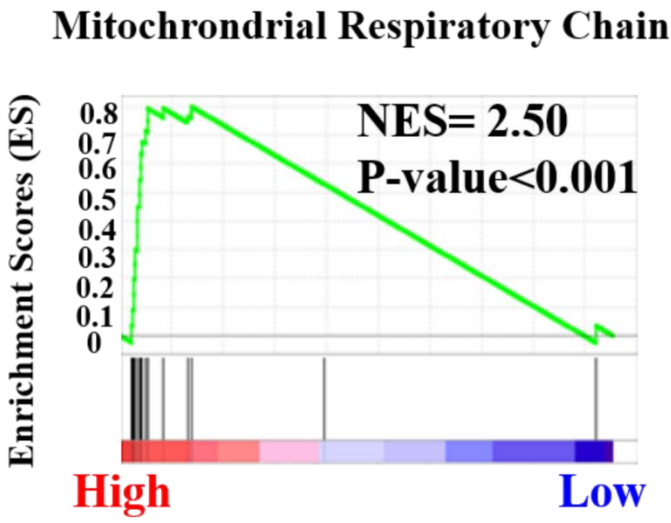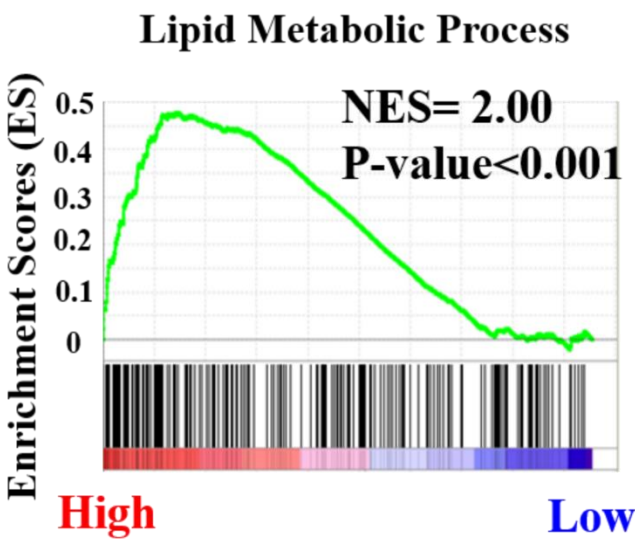

**B**

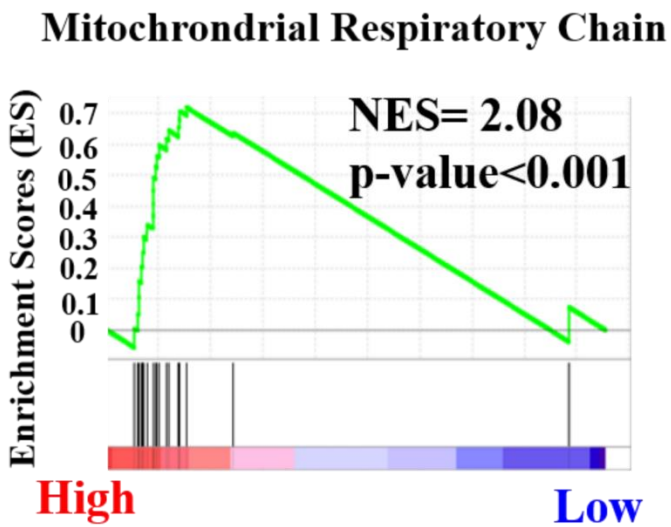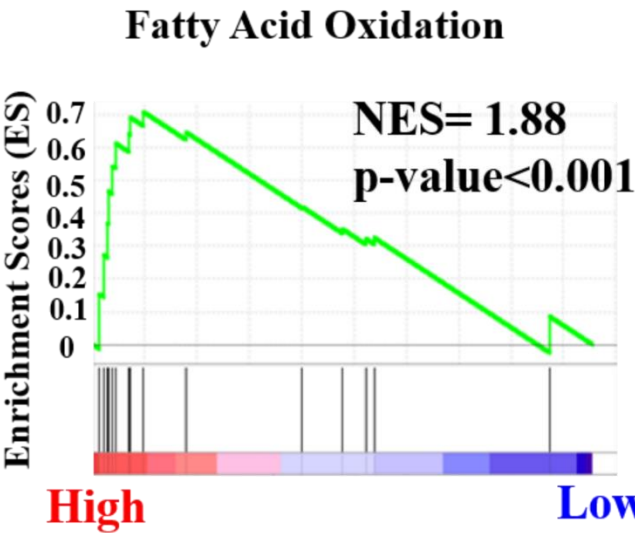

**Figure S3**

**A**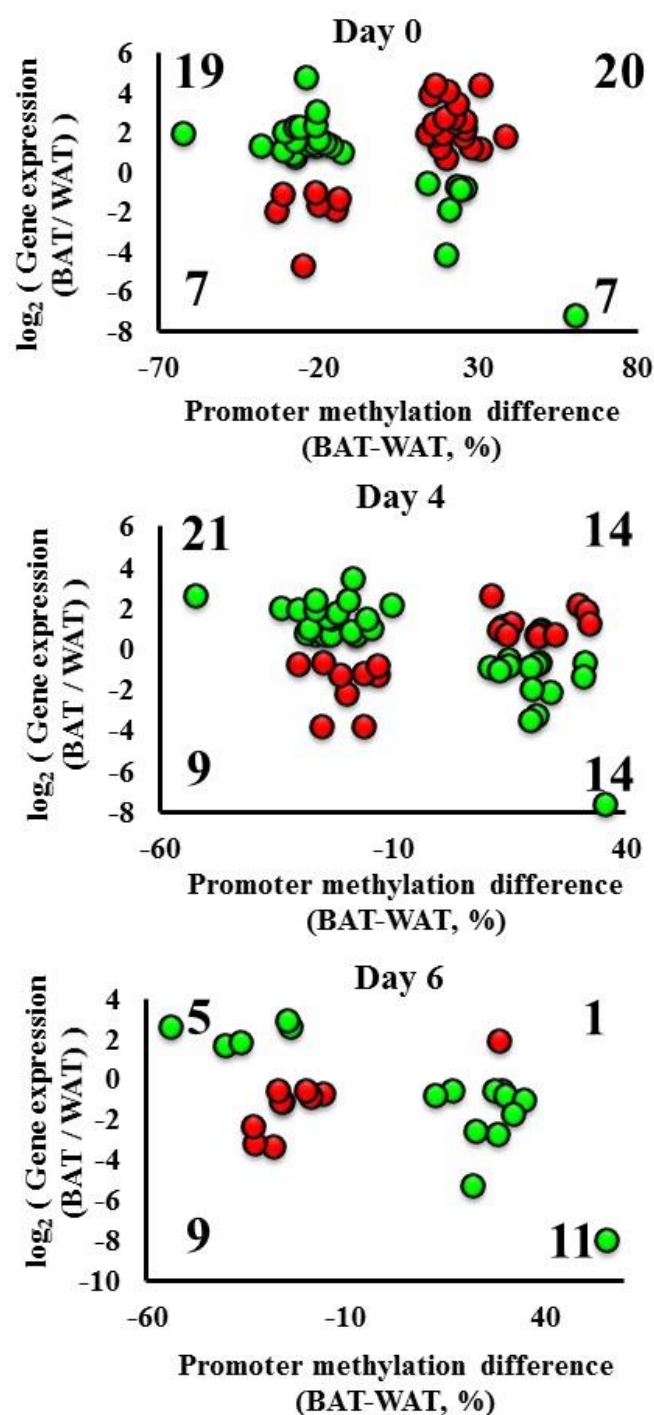

● Positive correlation

● Negative correlation

**B**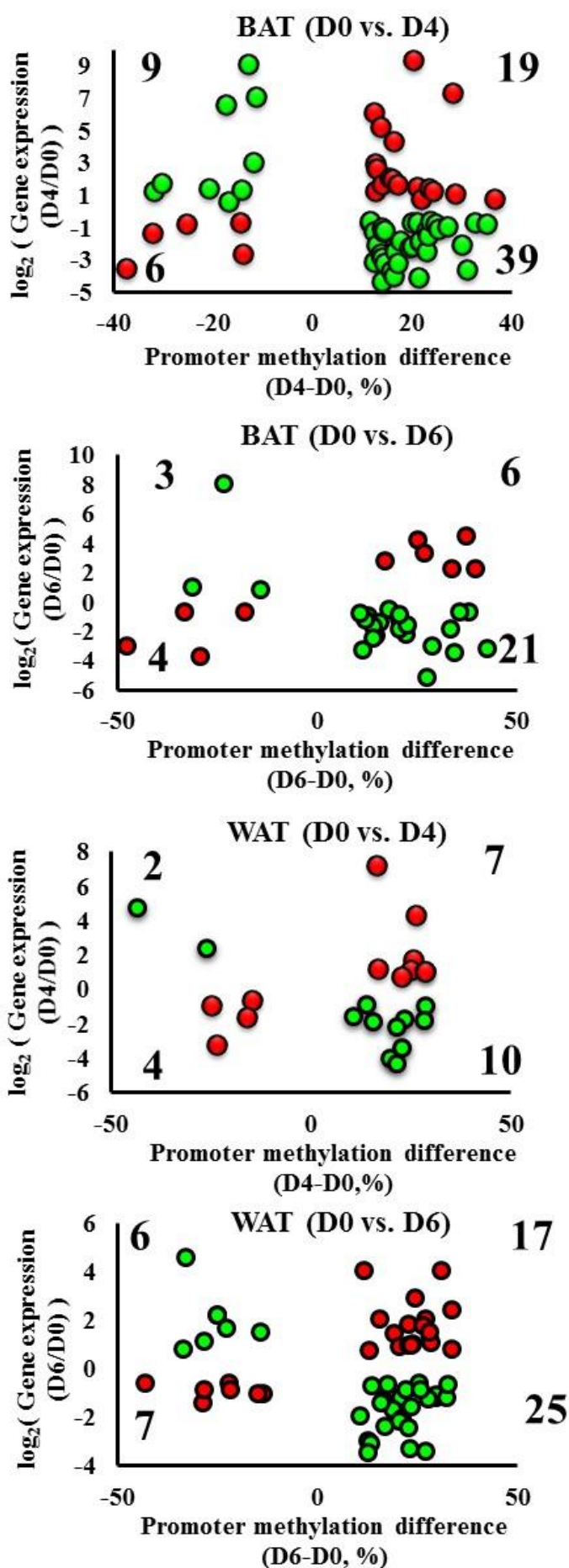**Figure S4**

Supplement: Supplementary file 2 [file mmc2.pdf]
